# LINC00460 mediates HMGA2 expression through binding to miRNA-143-5p competitively in gastric carcinoma

**DOI:** 10.55730/1300-0152.2648

**Published:** 2023-01-19

**Authors:** Xuqing ZHU, Yanli XIANG, Feifei MO, Lingling JIN

**Affiliations:** Department of Gastroenterology, Taizhou Municipal Hospital, Zhejiang, China

**Keywords:** LINC00460, miRNA-143-5p, HMGA2, gastric carcinoma, prognosis

## Abstract

**Background:**

Compelling evidence has manifested a strong association between aberrant expression of long noncoding RNAs (lncRNAs) and gastric carcinoma (GC) development. Nonetheless, biological impacts of differentially expressed lncRNAs (DElncRNAs) on GC are not scrutinized.

**Methods:**

Bioinformatics methods were employed for differential expression analysis and target gene prediction. MTT, colony formation, and Transwell methods were implemented for GC cell proliferation, migration, and invasion assessment. Western blot was implemented to test the protein level. The binding of genes was tested with dual-luciferase and RNA binding protein immunoprecipitation (RIP) approaches.

**Results:**

Noticeably high level of LINC00460 was observed in GC tissues and cells. LINC00460 silencing constrained proliferation, migration, and invasion of GC cells. FISH and nuclear-cytoplasmic separation assays confirmed the main presentation of LINC00460 in the cytoplasm. Bioinformatics predicted that LINC00460 had binding sites to miRNA-143-5p, which was upregulated in GC. Dual luciferase and RIP experiments also confirmed the binding relationship. Concurrent silencing of LINC00460s and miRNA-133-5p rescued the repressive influence of sh-LINC004600 on GC cell proliferation, migration, and invasion. HMGA2 was predicted to be a target gene downstream of miRNA-143-5p, their binding relationship was validated via dual luciferase assays. Silencing HMGA2 constrained GC cell proliferation, invasion, and migration. LINC00460 modulated HMGA2 expression via binding miRNA-143-5p, thereby affecting proliferation, invasion, and migration of GC cells.

**Conclusion:**

These findings validated that LINC00460 could regulate HMGA2 via sponging miRNA-143-5p to facilitate GC proliferation, invasion, and migration, which provides a deeper understanding of lncRNAs in the development of GC.

## 1. Introduction

Gastric carcinoma (GC) is a major global health proXXblem (Siegel et al., 2014). Early diagnosis is paramount for GC patients’ survival, but approximately two-thirds are diagnosed with advanced disease (Siegel et al., 2014). Despite the prominent progress in diagnosis and combination treatment, there is still no effective treatment available for improving the prognosis of patients with GC (Lazar et al., 2016). A great apprehension about corresponding mechanisms may inspire novel therapeutic and prognostic paths.

Numerous studies have manifested a strong correlation between lncRNAs and cancers. The aberrant expression of lncRNAs has been extensively recognized to be implicated in various tumorigenesis (Arun et al., 2018). As uncovered by Zhang and others, lncRNA MT1JP mediates FBXW7 via binding miRNA-92a-3p in GC (Zhang et al., 2018). Xu et al. illustrated that lncRNA AC130710 accelerates GC development via targeting miRNA-129-5p (Xu et al., 2014). LINC00460, a member of lncRNA, exerts an essential effect on tumor development (Sun et al., 2020). Hu et al*.* (2019) discovered that in nasopharyngeal carcinoma, LINC00460 accelerates invasion and metastasis through miRNA-30a-3p/Rap1A. Yuan et al*.* (2020) reported that LINC00460 fostered metastasis of colorectal cancer via targeting WWC2. In prostate cancer, LINC00460 inactivation represses cancer cell proliferation while stimulating cell apoptosis (Dong and Quan 2019). Cheng et al*.* (2021) manifested that LINC00460 fosters pancreatic cancer through modulating miR-320b/ARF1 axis. The discovery of these axes helps the prognosis and diagnosis. Activated LINC00460 expression in GC tissue acts as an independent prognostic marker (Yang et al., 2020). However, studies concerning this aspect remain scarce.

miRNAs possess broad basic biological activity and serve as transcriptional regulators for genes (Tao et al., 2017; Kurata et al., 2018). In several references, miRNAs have demonstrated their functions during GC development. The following are some typical examples in GC: miRNA-129-5p represses COL1A1 to mediate invasion and proliferation of cancer cells (Wang and Yu 2018). miRNA-122-5p restrains cancer cell migration and invasion via inactivating DUSP4 (Xu et al., 2018). miRNA-411 mediates SETD6 to repress cancer proliferation (Bai et al., 2019). Investigations focusing on miRNA-143-5p in GC growth are scarce.

Here, we identified a network of LINC00460/miRNA-143-5p/HMGA2 by using bioinformatics methods. To get more insight, in vitro experiments were conducted. Moreover, we also investigated the underlying mechanism of LINC00460 regulating HMGA2 through binding to miRNA-143-5p competitively in GC. Our investigation casts light on the pathogenesis of GC and helps in seeking therapeutic targets.

## 2. Materials and methods

### 2.1. Bioinformatics analysis

All GC-related data were accessed from The Cancer Genome Atlas (TCGA) (https://portal.gdc.cancer.gov/). R package “edge” (version 3.24.3) was implemented to conduct differential analysis of TCGA-derived GC-related expression profiles. The github repository for the code used in the analysis was https://github.com/732618078/ DERNA. The binding relationship between RNAs was predicted by TargetScan, miRWalk, and miRDB. Correlation analysis was done with Pearson’s correlation coefficient test.

### 2.2. Cell culture

GES (normal cells), HGC27, NCI-N87, and SNU-1 (GC cell lines) were provided by Cell bank of CAS (China). All cells were cultivated in Dulbecco’s Modified Eagle Medium (DMEM; sigma, USA) containing 10% fetal bovine serum (FBS; Gibco, USA). The cells were then maintained in an incubator with 5% CO_2_ at 37 °C.

### 2.3. Cell transfection

The sh-LINC00460, sh-HMGA2, and the corresponding negative control (sh-NC) vectors were constructed by Gene Pharma (China) using the pGPU6/GFP/Neo plasmid. The pcDNA3.1-HMGA2 (oe-HMGA2) and the corresponding blank control (oe-NC) were synthesized by Shanghai Sangon Biotech (China). miRNA-143-5p inhibitor, miRNA-143-5p mimic, and corresponding controls were accessed from RiboBio Co., Ltd. (China). Cells were transfected by lipofectamine 3000 (L3000015, Invitrogen, USA) per standard process. Sequences of miRNA-143-5p mimic and miRNA-143-5p inhibitor were as follows:

miRNA-143-5pmimic:
  5′-GGUGCAGUGCUGCAUCUCUGGU-3′
miRNA-143-5p inhibitor:
  5′-CCACGUCACGACGUAGAGACCA-3′

### 2.4. Real-time quantitative PCR (qRT-PCR)

Total RNA was separated from cancer cells by Trizol regent (Invitrogen, USA). Complementary DNA synthesis utilized reverse transfection kit (Invitrogen, USA). qRT-PCR was performed on ABI 7900HT instrument (Applied Biosystems, USA) with SYBR^®^ Primer Script TM RT-PCR Kit (Takara, Japan). All primers are presented in Table S1. U6 was utilized as an internal reference for miR-143-5p and β-actin was the internal reference for LINC00460 and HMGA2. The differential levels of target genes were assessed using 2^−ΔΔCt^ method.

### 2.5. Western blot

Cells were rinsed 3× cold PBS (Thermo Fisher, USA) after 48 h of transfection, and then were lysed on ice for 10 min. The protein concentration was tested with the BCA kit (Thermo Fisher, USA). Cell extracts were treated with SDS-PAGE at 100 V, and then separated proteins were transferred onto nitrocellulose membranes at 100 mA within 120 min. Membranes were maintained at 4 °C overnight with primary antibodies (rabbit anti-HMGA2, 1:1000, ab207301, Abcam, UK) after being blocked with 5% BSA/TBST. Next, 1× TBST (Solarbio, China) was used to rinse membranes on a shaking table 3 times (5 min/time) at room temperature. Horseradish peroxidase (HRP)-labeled secondary antibody goat antirabbit IgG (ab6721, Abcam, UK) was sequentially added for incubation for 120 min at room temperature. TBST was employed to rinse membranes 3 times, 20 min for each time. Internal reference protein was GAPDH (1: 1000, ab9485, Abcam, UK). All protein bands were visualized with ECL kit (Solarbio, China) and photographed. The experiment was conducted 3 times.

### 2.6. RNA Binding Protein Immunoprecipitation (RIP)

RIP kit (Millipore, USA) was utilized to conduct RIP. Lysate of HGC27 cells was cultured in RIP loading buffer with magnetic beads and incubated with human anti-Ago2 antibody (ab186733, Abcam, UK) with human IgG (ab7148, Abcam, UK) as control. Proteinase K was utilized for purification of the coimmunoprecipitated RNA for subsequent qPCR RNA. The coimmunoprecipitation samples were digested by proteinase K to extract detection.

### 2.7. MTT and colony formation methods

MTT was adopted for cell proliferation assessment. A total of 100 −L HGC27 or SNU-1 cells were plated into 96-well plates (5 **×** 10^3^ cells/100 −L). Each treatment underwent 3 repetitions. After being cultured for 24, 48, 72, 96, and 120 h, cell proliferative ability was assessed by using sterile MTT solution (Beyotime, China) per the protocol. Absorbance value at 570 nm was assessed by a microplate reader (Molecular Devices, USA).

For colony formation detection, HGC27 or SNU-1 cells were seeded into plates (1 **×** 10^3^ cells/well). After 14 days, the colonies were fixed in 30% formaldehyde for 15 min, and then stained with 0.1% crystal violet. The colonies (more than 50 cells) were counted by optical microscope.

### 2.8. Transwell assay

HGC27 or SNU-1 log phase cells were serum starved for 24 h. The next day, the cells were digested, centrifuged, and resuspended. For migration assay, the final concentration of the cells was 5 × 10^4^ cells/mL. A total of 0.2 mL cell suspension was added into Transwell upper chamber, with 700 −L DMEM + 10% FBS being filled into lower chamber. The cells were kept in an incubator with 5% CO_2_ at 37 °C. Twenty-four hours later, the cells in the upper chamber were removed with a wet cotton swab, while the cells migrating to the lower chamber were fixed in methanol for 30 min and stained with 0.5% crystal violet for 20 min.

For invasion assay, final concentration of the cells was 1 × 10^5^ cells/mL. The upper chamber was coated with Matrigel matrix (Corning, USA) and added with FBS-free DMEM. The lower chamber was filled with DMEM with 10% FBS (Thermo Fisher, USA). The cells were nurtured at 37 °C for 48 h, and the cells invading through Matrigel matrix were stained for 20 min with crystal violet, followed by PBS rinsing and drying in the two experiments. Five fields were randomly picked with a microscope to take photos and the cells were counted.

### 2.9. Dual-Luciferase detection

The psiCHECK2 luciferase reporter vectors (Promega, USA) ligated with wild-type (WT) and mutant-type (MUT) HMGA2/LINC00460 3′-untranslated-regions (3′UTR) were built, respectively. The psiCHECK2 vector was a polyclonal site that cloned the target gene to the downstream translation termination codon of renal luciferase, with firefly luciferase as the internal reference gene and renal luciferase as the main reporter gene. HGC27 cells (Thermo Fisher, USA) were seeded into 48-well plates for 24 h incubation. MiRNA-143-5p/NC and HMGA2/LINC00460 WT/MUT were cotransfected into cells. Finally, activity of luciferase was assayed by the luciferase assay kit (Promega, USA). Vector diagram of psiCHECK2 was shown in Figure S1.

### 2.10. Fluorescence in situ hybridization (FISH) and subcellular fractionation

For FISH assay, LINC00460 fluorescent probe was accessed from Ribo Co., Ltd. (China). According to instructions, Ribo Fluorescent in Situ Hybridization Kit and Ribo lncRNA FISH Probe Mix were employed for FISH assay (Ribo, China). Fluorescence microscope was utilized for observation and photographs. Nuclear-cytoplasm separation in HGC27 or SNU-1 cells was completed with PARIS Kit (Life Technologies, USA), and then qRT-PCR assayed levels of LINC00460 and reference genes (U6 and GAPDH) in nucleus and cytoplasm.

### 2.11. Statistics and analysis

Data were handled on GraphPad Prism 8.0 (GraphPad, USA). Measurement data were presented as mean ± standard deviation. Student’s *t*-test or one-way analysis of variance was employed for comparison between or among groups. p < 0.05 was of statistical significance.

## 3. Results

### 3.1. LINC00460 is activated in GC tissue and cells

**Figure 1:**
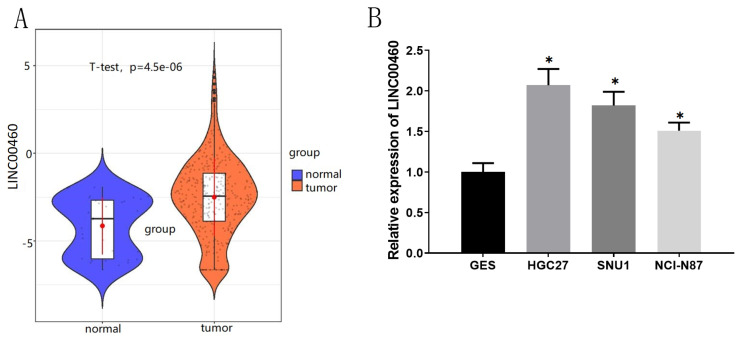
Expression of LINC00460 in tissue and cells. (A) LINC00460 expression in GC tissues predicted via TCGA; (B) The expression of LINC00460 in 3 GC cell lines and one normal GM cell line (GES);* p < 0.05.

LINC00460 is highly expressed in GC (Yang et al., 2020). We became interested in LINC00460 after reading this study; thus, we chose it as the research object. To investigate the expression of LINC00460 in GC, we conducted bioinformatics analysis and manifested that LINC00460 was notably upregulated in GC tissues (Figure 1A). Next, GES, HGC27, SNU1, and NCI-N87 cells were employed to validate LINC00460 expression. LINC00460 was markedly activated in GC cells (Figure 1B). Integrative conclusion was remarkably activated LINC00460 in GC. HGC27 and SNU1 cells bearing activated levels of this gene were next used in in vitro experiments.

### 3.2. LINC00460 silence represses migration, invasion, and proliferation of GC

**Figure 2:**
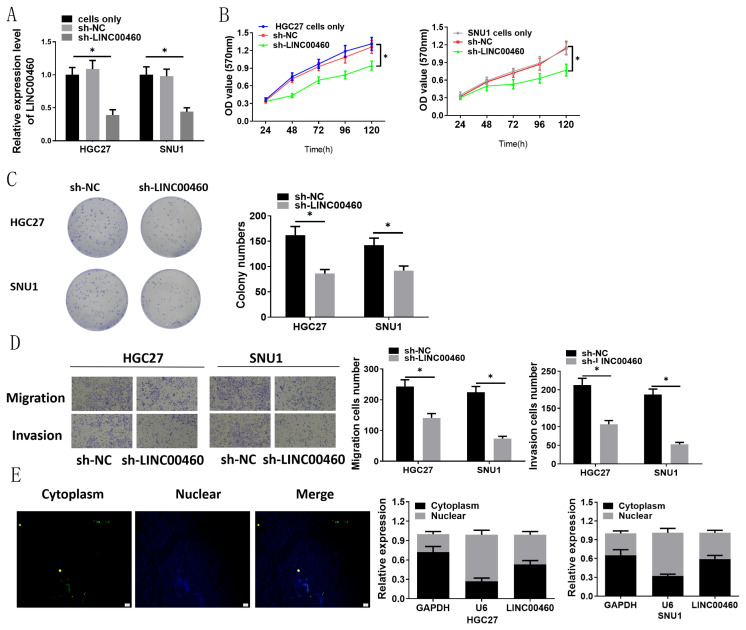
Sh-LINC00460 represses migration, invasion, and proliferation of GC cells, and LINC00460 mainly exists in cytoplasm. (A) Expression of LINC00460 in each cell groups was detected by qRT-PCR; (B, C, D) The viability, colony formation ability, migration and invasion of HGC27 and SNU-1 cells with silence of LINC00460 were detected by MTT assay, colony formation assay and Transwell assay (100×); (E) The LINC00460 expression location in GC tissue was tested by FISH and the expression levels of GAPDH (cytoplasmic marker), U6 (nuclear marker) and LINC00460 after the nucleus and cytoplasm of HGC27 and SNU-1 cells were separated by qRT-PCR; sh: Short hairpin; * p < 0.05.

LINC00460 expression is crucial for tumorigenesis of GC. We postulated that LINC00460 silence could repress migration, invasion, and proliferation. To confirm it, we examined the impacts of sh-LINC00460 on proliferation, migration, and invasion. Firstly, LINC00460 was silenced in HGC27 and SNU-1 cells, and LINC00460 level was assayed through qRT-PCR. Compared with untreated cancer cells and the control group, the cells transfected with sh-LINC00460 had significantly lower expression of LINC00460 (Figure 2A). The results of MTT and colony formation assays showed that sh-LINC00460 considerably restrained the cell viability and colony forming ability of HGC27 and SNU-1 cells (Figures 2B and 2C). Likewise, via transwell assay, cell migratory and invasive capabilities were evidently decreased upon LINC00460 suppression treatment (Figure 2D). These findings validated that LINC00460 could serve as an oncogene in GC cells. Additionally, FISH and nuclear-cytoplasm separation experiments verified that LINC00460 mainly existed in cytoplasm (Figure 2E). Overall, it could be understood that LINC00460 could promote the aggressive phenotypes of GC cells in cell cytoplasm.

### 3.3. LINC00460 is a sponge for miRNA-143-5p in GC and modulates GC cell proliferation, migration, and invasion via binding miRNA-143-5p

**Figure 3:**
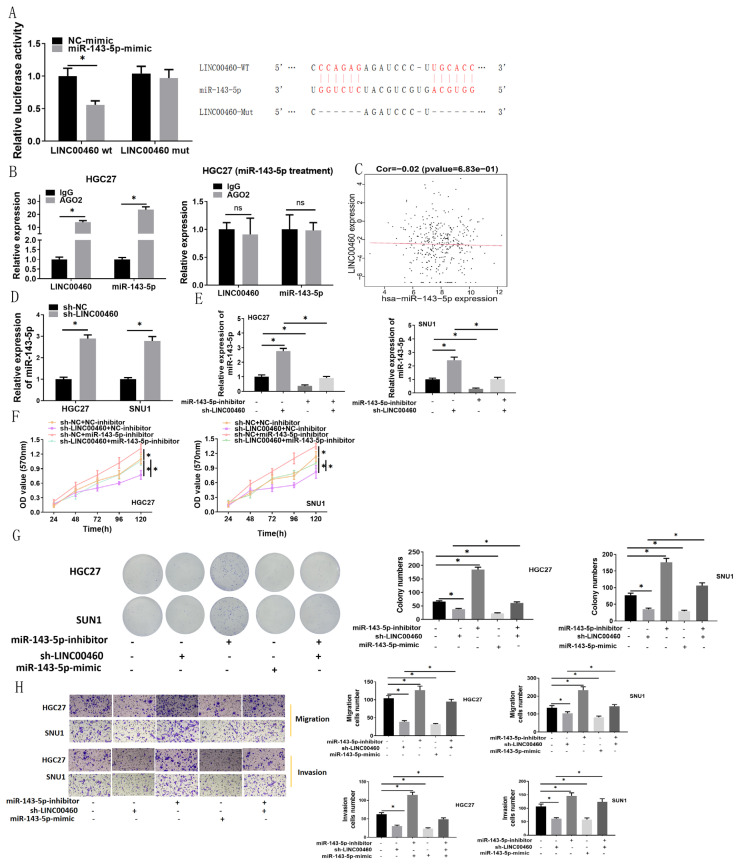
LINC00460 regulates the proliferation, migration, and invasion of GC cells by sponging miRNA-143-5p. (A) The binding relationship between LINC00460 and miRNA-143-5p in HGC27 cells was predicted and verified by miRDB database, dual-luciferase assay and RIP assay; (B) Binding relationship of LINC00460 with miRNA-143-5p in HGC27 cells as verified by RIP assay; (C) Correlation analysis of LINC00460 and miRNA-143-5p expression; (D) The relative expression of miRNA-143-5p in HGC27 and SNU-1 cell lines after overexpressing LINC00460 was detected by qRT-PCR; (E) Level of miRNA-143-5p in cells was detected by qRT-PCR; (F, G, H) The viability, colony formation ability, cell migration, and invasion of HGC27 and SNU-1 cells in different treatment groups were measured by MTT assay, colony formation assay and Transwell assay (100×); sh: Short hairpin; ns: Not significant; * p < 0.05.

We employed LncBase database to predict the binding site of LINC00460 to miRNA. It was found that LINC00460 had a binding site with miRNA-143-5p. Meanwhile, miRNA-143-5p is lowly expressed in GC tissues and cells (Shan et al., 2022). Since we were interested in miRNA-43-5p, it was utilized as the study gene. Dual-luciferase method was utilized to validate the targeting relationship of LINC00460 with miRNA-143-5p. The result manifested that miRNA-143-5p overexpression hindered luciferase activity of LINC00460-WT group, in the absence of impact on LINC00460-MUT group, suggesting the interaction between miRNA-143-5p and LINC00460 (Figure 3A). RIP experiments were performed based on HGC27 cell line to validate the binding relationship between miRNA-143-5p and LINC00460, in which the expression levels of LINC00460 and miRNA-143-5p were notably increased in AGO2 groups compared to IgG groups, whereas no notable differences between IgG and AGO2 groups were observed with the treatment of miRNA-143-5p inhibitor (Figure 3B). We assessed correlation of LINC00460 with miRNA-143-5p expression with the Pearson test. Results illustrated no correlation of LINC00460 with miRNA-143-5p expression (Figure 3C). Besides, miRNA-143-5p level was considerably increased upon silence of LINC00460 (Figure 3D). Altogether, LINC00460 was validated as a sponge of miRNA-143-5p.

Then, we conducted rescue experiments on GC cell lines HGC27 and SNU-1. SNU-1 experienced markedly increased miRNA-143-5p level after LINC00460 silence while attenuating the increasing trend after miRNA-143-5p silence (Figure 3E). As revealed by MTT assay, LINC00460 silence made a marked reduction in cell viability, while the impact was noticeably rescued when miRNA-143-5p and LINC00460 were concomitantly silenced (Figure 3F). In colony formation method, silencing of LINC00460 or overexpression of miRNA-143-5p substantially reduced the colony number, while the inhibitory effect was markedly relieved by silencing miRNA-143-5p and LINC00460 simultaneously (Figure 3G). The results of transwell assays presented that migration and invasion of GC cells were notably increased by silencing miRNA-143-5p, but miRNA-143-5p overexpression noticeably hampered these two cell behaviors, while the stimulatory effect was markedly relieved by inactivating miRNA-143-5p and LINC00460 simultaneously (Figure 3H). Collectively, these findings illustrated that LINC00460 may modulate GC cell proliferation, migration, and invasion by binding miRNA-143-5p.

### 3.4. HMGA2 is targeted by miRNA-143-5p

**Figure 4:**
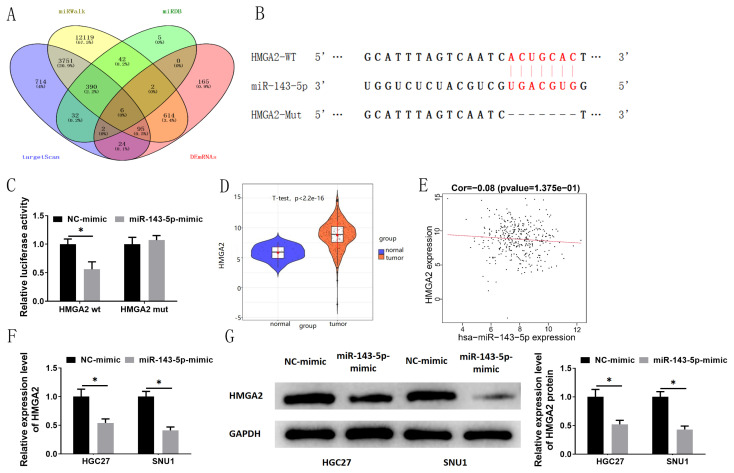
HMGA2 is targeted by miRNA-143-5p. (A) Venn diagram of genes; (B) The binding sites between HMGA2 and miRNA-143-5p were predicted by miRDB database; (C) The relationship between miRNA-143-5p and HMGA2 was verified by dual-luciferase assay; (D) HMGA2 expression in GC tissues was predicted via TCGA; (E) Correlation analysis of HMGA2 and miRNA-143-5p expression; (F, G) HMGA2 mRNA and protein expression levels in HGC27 and SNU-1 cells after treatment were assessed by qRT-PCR and western blot; * p < 0.05.

To investigate the genes downstream of miRNA-143-5p, bioinformatics analysis was done for prediction. Six mRNAs: HMGA2, AKAP4, ERP27, LIN28A, CLSPN, and XIRP1 were predicted targets downstream of miRNA-143-5p (Figure 4A). A growing number of studies have unveiled that HMGA2 is upregulated in varying cancers and mainly uses miRNAs as upstream targets to affect proliferation, migration, and invasion of multiple cancer cells, thus modulating cancer development (Gao et al., 2017; Zhao et al., 2018; Xia et al., 2021). Based on the previous studies, we considered whether HMGA2 could bind to miRNA-143-5p. MiRDB database was employed for predicting binding site of the two genes. The result showed the predicted binding sites of miRNA-143-5p on HMGA2 3′UTR (Figure 4B). Dual-luciferase method results illustrated that overexpression of miRNA-143-5 reduced the fluorescence activity of HMGA2-wt, indicating that HMGA2 was targeted by miRNA-143-5p (Figure 4C). Bioinformatics method revealed a substantial upregulation of HMGA2 in GC (Figure 4D). Pearson correlation analysis illustrated no correlation in expression of HMGA2 with miRNA-143-5p (Figure 4E). To investigate whether miRNA-143-5p affects HMGA2 in GC, HMGA2 mRNA and protein expressions were assayed via qRT-PCR and western blot with miRNA-143-5p overexpression. The results presented that upregulation of miRNA-143-5p repressed HMGA2 mRNA and protein expressions compared with the control group (Figures 4F and 4G). To sum up, HMGA2 was targeted by miRNA-143-5p, and miRNA-143-5p modulated HMGA2 expression in GC cells.

### 3.5. Lower expression of HMGA2 represses migration, invasion, and proliferation of GC cells

**Figure 5:**
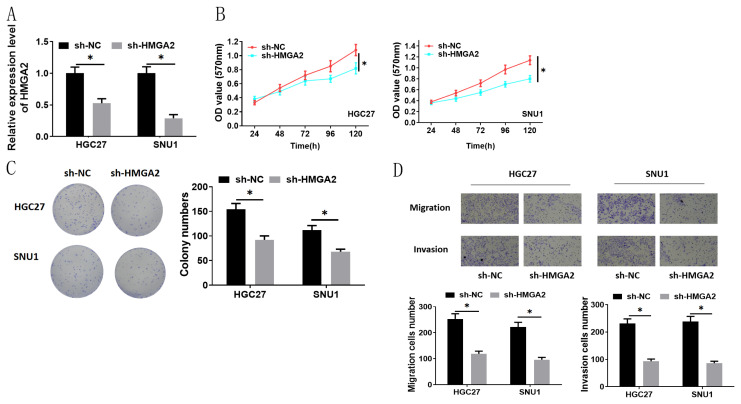
Lower expression of HMGA2 represses the proliferation, migration, and invasion of GC cells. (A) Level of HMGA2 in each cell group was detected by qRT-PCR; (B, C, D) The cell viability, cell growth, migration and invasion of HGC27 and SNU-1 cells after silencing HMGA2 were assessed by MTT, cell colony formation and Transwell methods (100×); sh: Short hairpin; * p < 0.05

To determine HMGA2 involved in migration, invasion, and proliferation in GC cells, qRT-PCR was utilized to assay transfection efficiency. Results illustrated that HMGA2 level was substantially reduced after silencing HMGA2, indicating a good transfection efficiency (Figure 5A). MTT and colony formation assays both revealed that silencing HMGA2 repressed viability and cell colony formation ability of HGC27 and SNU-1 cells (Figures 5B and 5C). In the meantime, cell migration and invasion assays indicated that silencing HMGA2 inhibited cell migratory and invasive abilities of HGC27 and SNU-1 cells as well (Figure 5D). Overall, HMGA2 acted as a cancer promoter to facilitate the proliferation, migration, and invasion of GC cells.

### 3.6. LINC00460 promotes HMGA2 expression by inhibiting miRNA-143-5p

**Figure 6:**
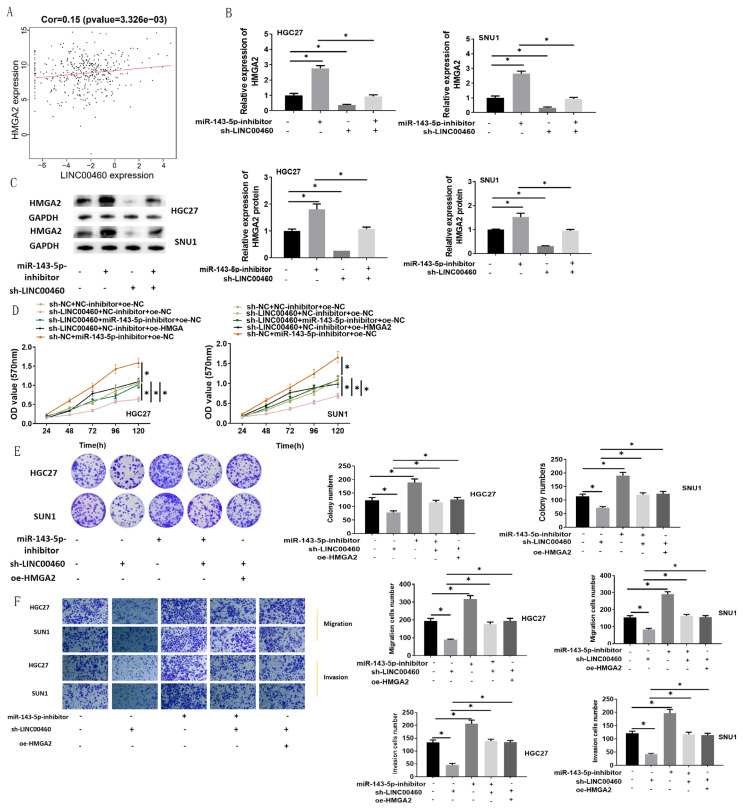
LINC00460 facilitates HMGA2 expression by suppressing miRNA-143-5p. (A) Correlation analysis of LINC00460 and HMGA2 expression; (B) The mRNA expression of HMGA2 in HGC27 and SNU-1 cell lines upon silence of LINC00460 and miRNA-143-5p as detected by qRT-PCR; (C) The protein expression of HMGA2 in HGC27 and SNU-1 cell lines upon silence of LINC00460 and miRNA-143-5p was detected by western blot; (D, E, F) The viability, colony formation ability, cell migration and invasion of HGC27 and SNU-1 cells in different treatment groups were measured by MTT assay, colony formation assay, and Transwell assay (100×); oe: Overexpression; sh: Short hairpin; * p < 0.05.

Then we explored whether LINC00460 affected GC cells through the miRNA-143-5p/HMGA2 axis. Pearson correlation analysis revealed a positive correlation of LINC00460 with HMGA2 (Figure 6A). By analyzing the protein expression and mRNA expression of HMGA2 upon silence of LINC00460 and miRNA-143-5p at cellular level, we found that LINC00460 silence repressed HMGA2 protein expression and mRNA expression while miRNA-143-5p silence facilitated HMGA2 protein expression and mRNA expression (Figure 6B–C). MTT, colony formation, and Transwell assays were introduced to examine GC cell proliferative, migratory, and invasive properties. The results presented that miRNA-143-5p-inhibitor fostered proliferative, migratory, and invasive properties of GC cells, but LINC00460 silencing repressed these properties. Cell proliferation, migratory and invasive capacities attenuated by LINC00460 silencing can be rescued by inhibiting miRNA-143-5p or upregulating HMGA2 (Figures 6D–6F). In summary, LINC00460 may regulate HMGA2 to modulate the proliferation, migration, and invasion of GC cells by competitively binding to miRNA-143-5p. Hence, the findings suggested that LINC00460/ miRNA-143-5p/ HMGA2 affected GC cell proliferation, migration, and invasion.

## 4. Discussion

Despite compelling evidence, how LINC00460 mediates GC development remains incompletely described (Zhang et al., 2019). We revealed high levels of LINC00460/HMGA2 while low level of miRNA-143-5p in GC cells. In vitro experiments manifested promoting impacts of LINC00460 on GC development via sponging miRNA-143-5p. All in all, LINC00460 repressed miRNA-143-5p to mediate HMGA2, thereby impacting GC proliferation, invasion, and migration.

Tregs-related competing endogenous RNA (ceRNA) networks are strongly associated with the development of multiple cancers (Wu et al., 2020; Chen et al., 2021; Liu et al., 2021; Ma et al., 2021). Thus, ceRNA network has a pivotal role in cancer prognosis and treatment. To dig out new GC therapeutic markers, it is key to investigate the role and mechanism of the lncRNA/ miRNA/ mRNA axis in GC genesis. Here, the focus was the specific regulatory role of LINC00460/miRNA-143-5p/HMGA2 axis in GC. Our results showed that overexpressing LINC00460 had a promoting effect on migration, invasion, and proliferation of GC cells. These impacts were also mentioned in other references but in different cancers. LncRNAs are strongly associated with cancer progression. In colon cancer (Wu et al., 2018; Hong et al., 2020), nonsmall cell lung cancer (Wang et al., 2020), papillary thyroid cancer (Li and Kong 2019), and cervical cancer (Li et al., 2021), lncRNAs act as competing endogenous RNAs mainly by suppressing miRNA-targeting-mRNA and modulating cancer cell proliferation, migration, and invasion, thereby affecting cancer progression. In this study, our investigation of the impacts of LINC00460 on GC cell migration, invasion, and proliferation further confirmed its regulatory role in cancer progression by serving as a tumor promoter. In terms of the interaction among LINC00460, miRNA-143-5p and HMGA2, we found that LINC00460 competitively binding to miRNA-143-5p with HMGA2 to regulate HMGA2 expression to affect the proliferation, migration, and invasion of GC cells. Jin et al. manifested that lncRNA TCONS-00026907 mediates development of cervical cancer by repressing miRNA-143-5p expression (Jin et al., 2017). Chen et al. described a prominent correlation between COL5A2 expression and prognosis of tongue squamous cell carcinoma (Chen et al., 2019). Here, we verified that miRNA-143-5p as a tumor repressor hindered GC progression when it was overexpressed. HMGA2 was found to be a cancer promoter, and silencing HMGA2 could constrain GC progression. These findings elucidate effects of miRNA-143-5p and HMGA2 in the LINC00460/ miRNA-143-5p/HMGA2 axis on GC cell proliferation, invasion, and migration.

In conclusion, the investigation elucidated that the LINC00460/miRNA-143-5p/HMGA2 axis was tightly linked to GC progression, and data suggested that LINC00460 exerted positive modulatory role in GC cells. Besides, LINC00460 could regulate HMGA2 to facilitate functions of GC cells by sponging miRNA-143-5p. These findings cast light on novel targeted therapies of GC. Limitations exist such as no more investigation of in vivo experiments and clinical samples were also necessary.

## Supplementary materials

Figure S1 Vector diagram of psiCHECK2.

**Table S1:** Primer sequences used in the study.

Gene	Sequence	
miRNA-143-5p	Forward primer	5′- GGTGCAGTGCTGCATCTCTGGT -3′
LINC00460	Forward primer	5′- CAGGGGGACTCATCTCCTCA-3′
	Reverse primer	5′- CCATTTCAGAGGCGTGGACT-3′
HMGA2	Forward primer	5′- CAGCAAGAACCAACCGGTGA-3′
	Reverse primer	5′- TCCCAGGCAAGGCAACATTGA-3′
β-actin	Forward primer	5′-TCGTGCGTGACATTAAGGAG-3′
	Reverse primer	5′-GTCAGGCAGCTCGTAGCTCT-3′
U6	Forward primer	5′-CTCGCTTCGGGCAGCACA-3′
	Reverse primer	5′-AACGCTTCACGAATTTGCGT-3′

## Data Availability

The data and materials in the current study are available from the corresponding author on reasonable request.
