# Supplementary figures and images for: LINC00460 mediates HMGA2 expression through binding to miRNA-143-5p competitively in gastric carcinoma
Source: Turk J Biol. 2023 Jan 19;47(2):130–40. doi: 10.55730/1300-0152.2648 (PMC10387914; doi:10.55730/1300-0152.2648)

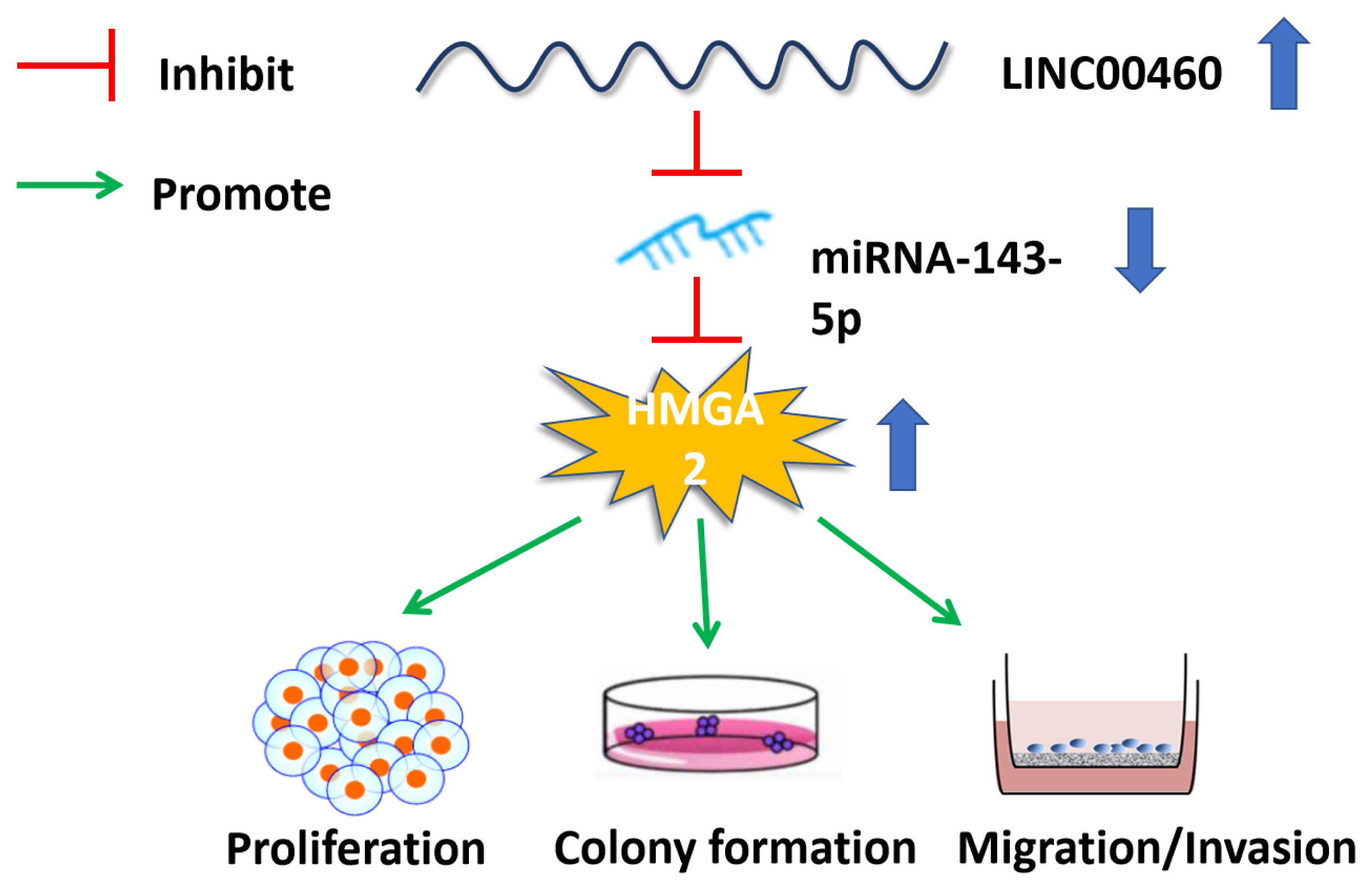

Supplement: Figure S1 — Vector diagram of psiCHECK2. [file turkjbiol-47-2-120s1.tif]
